# Preliminary study of Gd-EOB-DTPA contrast-enhanced magnetic resonance imaging for determining gross tumor volume in hepatocellular carcinoma radiotherapy

**DOI:** 10.3389/fonc.2025.1720806

**Published:** 2026-01-22

**Authors:** Kangning Meng, Guanzhong Gong, Ruozheng Wang, Yong Yin

**Affiliations:** 1 Department of Radiology, Shandong First Medical University Affiliated Cancer Hospital, Shandong Cancer Hospital and Institute (Shandong Cancer Hospital), Jinan, China; 2 Department of Radiation Physics, Shandong First Medical University Affiliated Cancer Hospital, Shandong Cancer Hospital and Institute (Shandong Cancer Hospital), Jinan, China; 3 Radiotherapy Cancer, Affiliated Cancer Hospital of Xinjiang Medical University, Urumqi, China

**Keywords:** CE-MRI, Gd-EOB-DTPA, gross tumor volume determination, hepatocellular carcinoma, radiotherapy

## Abstract

**Purpose:**

The aim of this study was to evaluate the feasibility of gadolinium ethoxybenzyl diethylenetriamine pentaacetic acid (Gd-EOB-DTPA) contrast-enhanced magnetic resonance imaging (CE-MRI) for determining the gross tumor volume (GTV) of hepatocellular carcinoma (HCC).

**Methods:**

A retrospective analysis was conducted on 12 patients diagnosed with HCC (18 lesions) who received radiotherapy and underwent magnetic resonance (MR) simulation. Six series images, including MR T_1_-weighted image (T_1_WI) and contrast-enhanced T_1_WI (CE-T_1_WI) at 15 s, 45 s, 75 s, 150 s, and >20 min after Gd-EOB-DTPA injection, were obtained, and the GTV was determined in the different temporal images. The differences in mean signal intensity (SI), SI contrast between the HCC and liver tissue, volume and shape of HCC GTV among different phases were compared.

**Results:**

(1) The mean SI of liver tissue reached its peak enhancement at >20 min, showing a 140.90 ± 64.69% increase, compared with T_1_WI (*p* < 0.05). (2) Compared with CE-T_1_WI_-20min_, the mean SI of the HCC increased by -41.19~18.09% from T_1_WI, CE-T_1_WI_-15s_ to CE-T_1_WI_-150s_. Conversely, the mean SI of liver tissue decreased by 5.27~55.87% over the same period. Consequently, the SI contrast between HCC and liver tissue decreased by 53.30~89.37%. (3) The maximum GTV volume determined by CE-T_1_WI_-20min_ was (22.80 ± 18.57) cm^3^, coinciding with the highest value of SI contrast (0.29 ± 0.16). (4) Compared with GTV_-20min_, GTV_-T1WI_ and GTV_-15s_~GTV_-150s_ had volume reductions of 6.73~19.35%. (5) Compared with GTV_-20min_, the Dice similarity coefficients (DSC) of GTV_-T1WI_ and GTV_-15s_~GTV_-150s_ ranged from 0.745 to 0.819. Additionally, the shape change trend of GTV in the CE-T_1_WI images was generally consistent with the volume change trend.

**Conclusion:**

CE-T_1_WI MR images acquired more than 20 min post-injection of Gd-EOB-DTPA exhibited significant advantages in determining the GTV boundaries and enhancing the contrast of SI between HCC and liver tissue. The CE-T_1_WI_-20min_ sequence is recommended for determining HCC GTV.

## Introduction

Hepatocellular carcinoma (HCC) is one of the most prevalent malignant tumors and ranks fourth among the causes of tumor-related deaths (1). The 5-year survival rate for patients with HCC is less than 20% (2). Precision radiotherapy has become indispensable for the comprehensive treatment of HCC, significantly improving the efficacy of radiotherapy while reducing the incidence of radiation-induced liver disease (RILD) (1). Notably, accurate determination of gross tumor volume (GTV) is essential to ensure the accuracy and efficacy of radiotherapy for HCC (3).

With a high soft-tissue resolution, magnetic resonance imaging (MRI) offers a clear determination of the HCC boundaries (4). Extracellular agents (ECAs) are commonly used in clinical practice for contrast-enhanced magnetic resonance imaging (CE-MRI) of HCC due to their capability to achieve a high contrast between the tumor and liver tissue in the arterial phase, which is useful when detecting HCC. However, the contrast is reduced in the delayed phase, which poses challenges for GTV determination (5). Gadolinium ethoxybenzyl diethylenetriamine pentaacetic acid (Gd-EOB-DTPA) is a hepatobiliary-specific MR contrast agent that combines the characteristics of ECAs with those of hepatobiliary-specific contrast agents (6). Notably, hepatobiliary phase imaging showcases heightened liver tumor contrast and clear tumor boundaries (6).

However, few reports have explored the determination of HCC GTV using Gd-EOB-DTPA CE-MRI, which highlights the need for in-depth feasibility studies. Therefore, this study aimed to provide a basis for determining the GTV of HCC based on Gd-EOB-DTPA by quantitatively analyzing the differences in HCC imaging using different temporal CE-MRI sequences.

## Materials and methods

### Patient information

A retrospective study was conducted on 12 patients with HCC (18 lesions) who received radiotherapy at Shandong First Medical University Affiliated Cancer Hospital between January and October 2023. The mean age was 61.17 years (range: 53~77 years). The cohort included nine males (14 lesions) and three females (4 lesions). Detailed information of the patients is shown in **Table 1**.

**Table 1:** Basic patient information.

n (%) Total n=12	
Age (years)	
Mean (range)	61.17 (53-77)
Sex	
Male	9 (75%)
Female	3 (25%)
Liver cirrhosis	
Yes	12 (100%)
No	0 (0%)
Child-Pugh Classification	
Child-Pugh Class A	12 (100%)
Child-Pugh Class B/C	0 (0%)
Delayed scanning (20 min)	
Mean specific time (min, range)	20.75 (19~27)

The inclusion criteria were listed as follows: (1) confirmation of HCC by pathological biopsy, (2) absence of contraindications to MR, (3) without radiotherapy previously, and (4) availability of MR contrast-enhanced T_1_-weighted imaging (CE-T_1_WI) enhanced at 15 s, 45 s, 75 s, 150 s, and 20 min post-contrast agent injection. Ethical approval for this study was obtained from the Ethics Review Committee of Shandong Cancer Hospital (Ethics approval number: SDTHEC201903032), and all patients provided signed informed consent.

#### MR simulation

**Figure 1:**
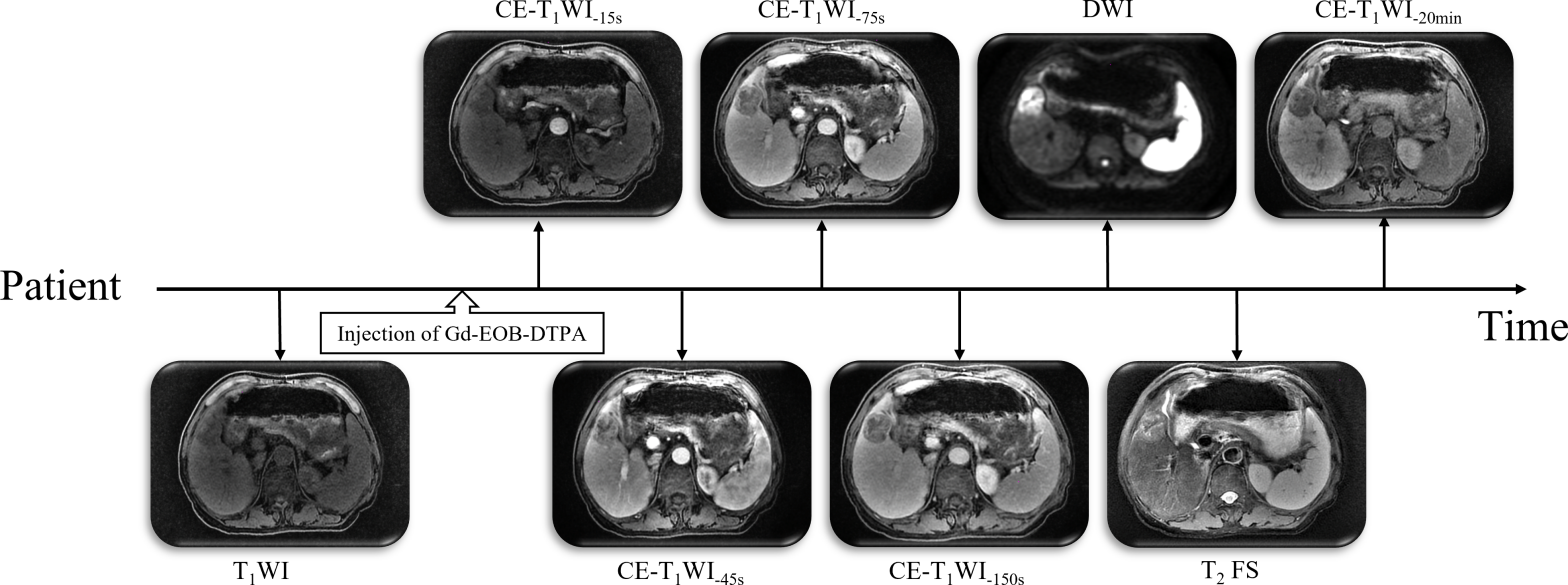
MR simulation positioning sequence scanning process. DWI, diffusion-weighted imaging; T_2_ FS, T_2_-weighted imaging with fat suppression.

All patients were fixed in a vacuum-log bag, in a supine position with their arms above their heads, and MR simulation localization was performed on a GE 3.0T superconducting MR scanner (Discovery 750W, GE Healthcare, Chicago, IL, USA). The scanning range was from 3–4 cm above the diaphragm to the lower pole of the right kidney (7). Six sequences of scanning were performed in sequence: the pre-contrast T_1_-weighted images (T_1_WI) scan and CE-T_1_WI scan at 15 s, 45 s, 75 s, 150 s, and >20 min after intravenous injection of Gd-EOB-DTPA (Primovist; Bayer AG, Berlin, Germany). The CE-T_1_WI sequences were labeled as CE-T_1_WI_-15s_, CE-T_1_WI_-45s_, CE-T_1_WI_-75s_, CE-T_1_WI_-150s_, and CE-T_1_WI_-20min_, respectively. Additionally, the pre-contrast T_1_ scan is hereinafter referred to as T_1_WI; the scanning process is shown in **Figure 1**.

The MR scanning sequence parameters were as follows: TR = 5.2 ms, TE = 2.7 ms, FOV = 42~50 cm, matrix = 296 × 256 mm, slice thickness = 3.0 mm, slice spacing= 0 mm, with end-expiration breath holding (EEBH) respiratory state. The CE-T_1_WI sequence was administered with an MR injection system (MEDRAD@ Spectris Solaris EP, Bayer, Leverkusen, Germany) at a dose of 0.1 mL/kg and an injection rate of 1 mL/s. Following the Gd-EOB-DTPA injection, a 20 mL flush of normal saline was administered.

#### HCC GTV determination, naming, and registration

**Figure 2:**
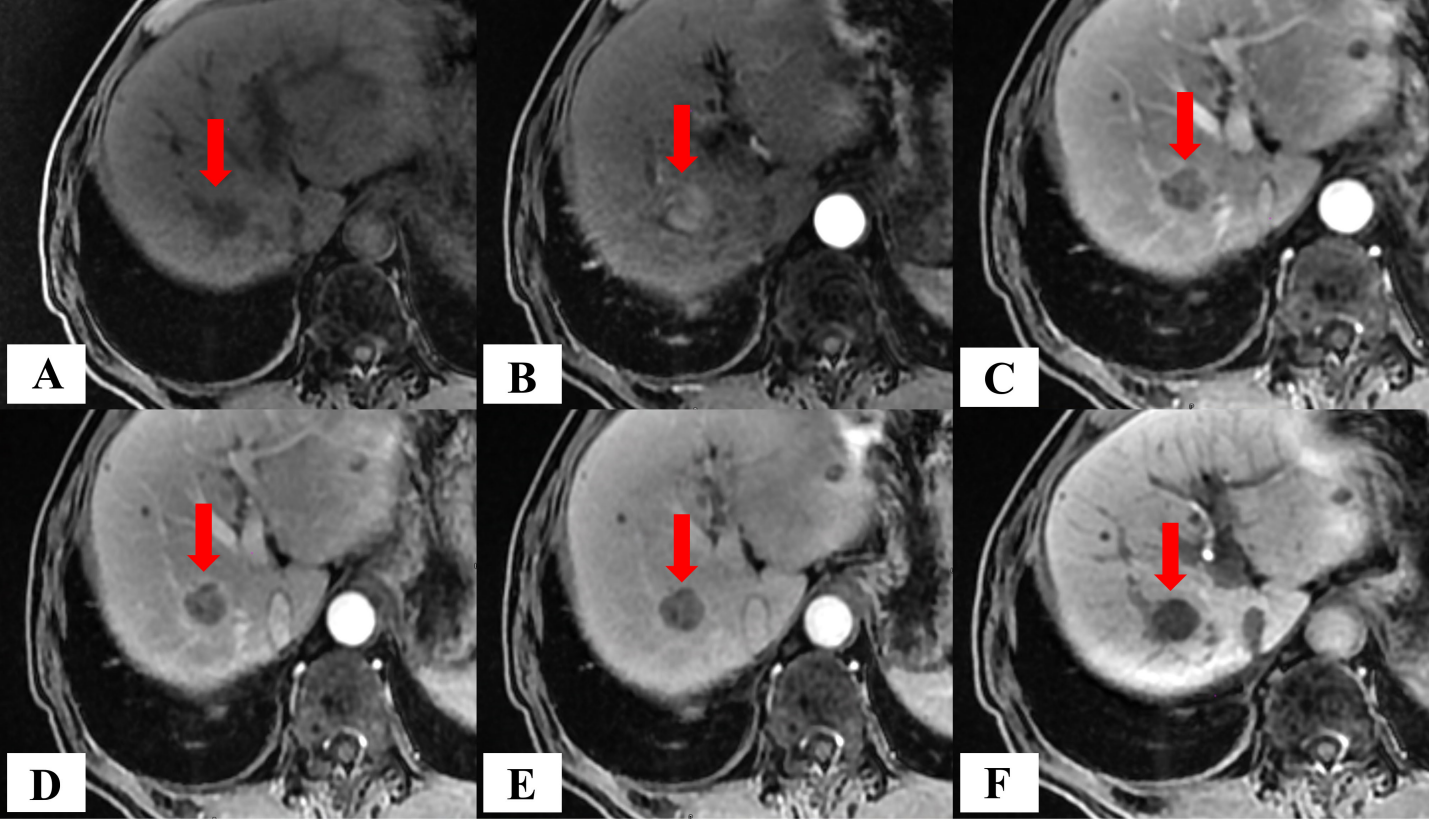
Schematic diagram of MRI images across different phases in a patient with HCC. **(A)** T_1_WI; **(B–F)** CE-T_1_WI acquired at 15 s, 45 s, 75 s, 150 s, and 20 min post-intravenous injection of Gd-EOB-DTPA, respectively.

The lesion was defined as a mass with low-to-medium signal intensity (SI) on T_1_WI, exhibiting high SI at CE-T_1_WI_-15s_ and relatively low SI from CE-T_1_WI_-45s_ to CE-T_1_WI_-20min_ (**Figure 2**).

All MR images were imported into MIM Maestro software (version 7.1.7, Cleveland, OH, USA). The T_1_WI sequence images were selected as the primary sequence for registration, and the remaining five imaging sequences were rigidly registered to the T_1_WI sequence. A radiation oncologist manually determined the GTV on T_1_WI, CE-T_1_WI_-15s_~CE-T_1_WI_-20min_ sequences. This determination was subsequently reviewed and modified by two additional radiation oncologists. In cases of disagreement, the three doctors discussed and reached a consensus on the GTV boundaries. The GTVs were labeled sequentially as GTV_-T1WI_, GTV_-15s_, GTV_-45s_, GTV_-75s_, GTV_-150s_, and GTV_-20min_. In addition, a 1 cm^3^ volume of liver tissue, locating 2 cm outside the lesion and avoiding the large hepatic artery, hepatic vein, portal vein, and bile duct system, was designated as liver tissue.

#### GTV information acquisition

Statistical analysis encompassed the mean SI and SI contrast of the HCC GTV and liver tissue, as well as the volumes of all GTVs. Additionally, comparisons were conducted between the GTV_-20min_ and other GTVs of Dice similarity coefficient (DSC), volume differences, and volume reduction rates. The SI contrast was calculated using Equation 1, whereas the DSC is defined by Equation 2. Equation 3 was used to determine the rate of volume reduction.

(1) $$SI\;contrast=\frac{\left|SI_{HCC}-SI_{liver\;tissue}\right|}{SI_{liver\;tissue}}$$

(2) $$DSC=\frac{2(|GTV_{-x}|\cap |GTV_{-20\min}|)}{\left|GTV_{-x}|+|GTV_{-20\min}\right|}$$

(3) $$volume\;reduction\;rate=\frac{GTV_{-20\min}-GTV_{-x}}{GTV_{-20\min}}$$

Note: GTV_-x_ represents a certain sequence of GTV, and SI_HCC_ represents the HCC SI. SI_liver tissue_ represents liver tissue SI.

### Statistical analysis

Statistical analyses were conducted using IBM SPSS statistical software (version 25.0, Armonk, NY, USA). The Wilcoxon test was used to analyze the differences in the mean SI, SI contrast of the GTVs and liver tissue, and GTV volumes. Statistical significance was set at *p* < 0.05.

## Results

### Comparison of the mean SI between GTV and liver tissue across different phases

#### Comparison of the mean SI values of GTVs across different phases

**Figure 3:**
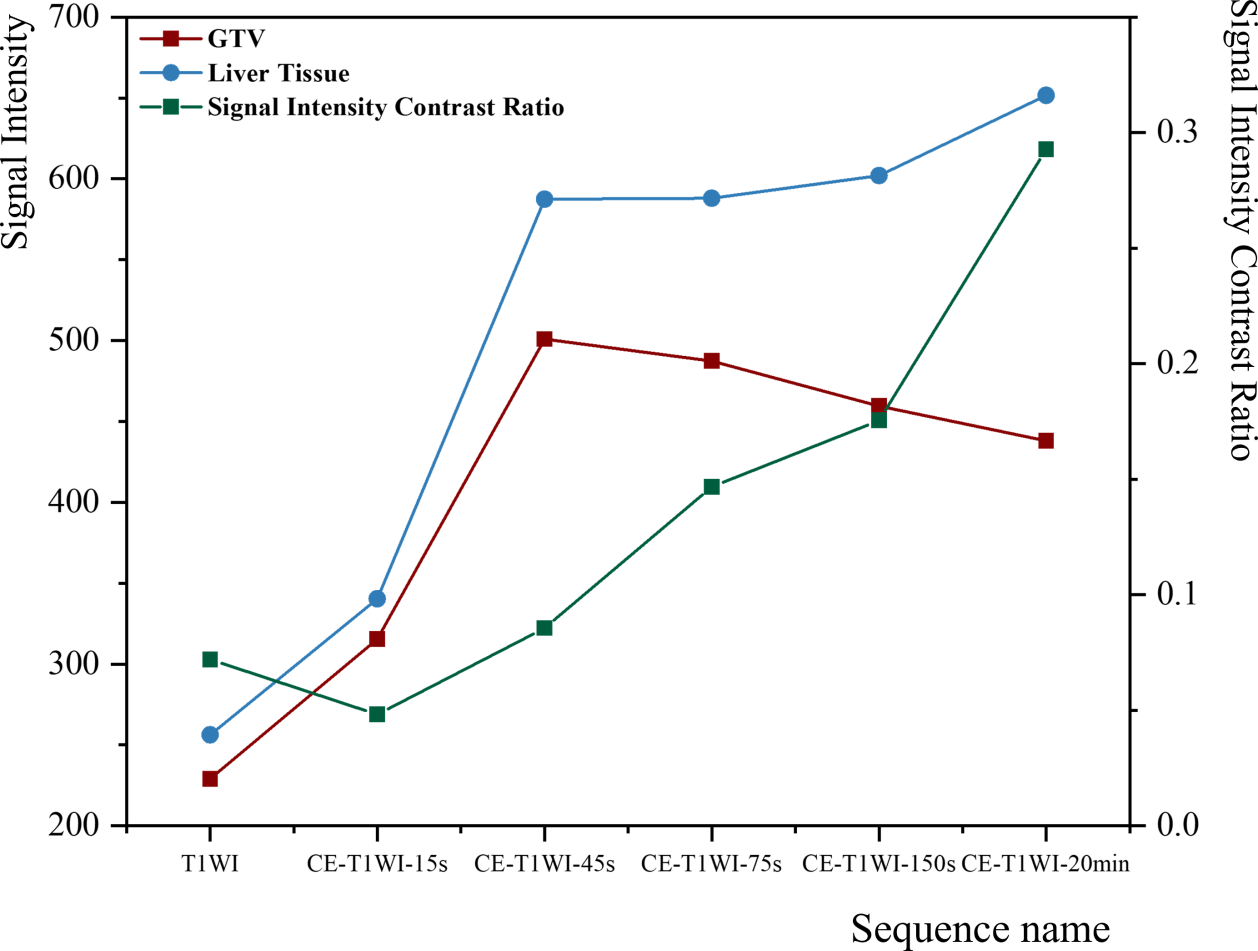
Trends in the mean SI of liver tissue and HCC GTV and SI contrast across different phases.

The mean SI of the GTVs exhibited a trend of initially increasing and then gradually decreasing across the different phases. The most significant increase was observed from GTV_-15s_ to GTV_-45s_ (**Figure 3**). Among the different phases, the mean SI of GTV_-45s_ was the highest (501.01 ± 229.11), whereas GTV_-T1WI_ exhibited the lowest mean SI at 229.06 ± 68.22 (**Table 2**).

**Table 2:** Summary table of mean SI and SI contrast between HCC and liver tissue across different phases.

Sequence name	Mean SI						SI contrast		
	GTV			Liver tissue					
	Value	Difference (%)	*p*	Value	Difference (%)	*p*	Value	Difference (%)	*p*
T_1_WI	229.06 ± 68.22	-41.19 ± 19.74	0.000	256.21 ± 88.29	-55.87 ± 10.84	0.000	-0.07 ± 0.22	-80.70 ± 104.97	0.000
CE-T_1_WI_-15s_	315.47 ± 156.78	-23.94 ± 28.70	0.002	340.35 ± 175.96	-45.23 ± 14.08	0.000	0.05 ± 0.19	-85.41 ± 88.90	0.000
CE-T_1_WI_-45s_	501.01 ± 229.11	18.09 ± 24.25	0.028	587.53 ± 305.38	-7.99 ± 19.17	0.019	-0.09 ± 0.24	-89.37 ± 168.22	0.001
CE-T_1_WI_-75s_	487.35 ± 218.93	14.61 ± 21.65	0.039	588.09 ± 278.77	-5.27 ± 17.25	0.015	-0.15 ± 0.18	-57.24 ± 79.65	0.000
CE-T_1_WI_-150s_	459.58 ± 207.48	7.16 ± 13.94	**0.133**	602.09 ± 274.48	-7.42 ± 17.12	0.003	-0.18 ± 0.19	-53.30 ± 57.57	0.001
CE-T_1_WI_-20min_	438.02 ± 201.59	\	\	651.64 ± 357.75	\	\	-0.29 ± 0.16	\	\

Difference indicates the rate of change relative to CE-T_1_WI_-20min_, where a positive value represents an increase and a negative value represents a decrease. The *p-*value represents the result of the Wilcoxon test comparing other sequences with CE-T_1_WI_-20min_.

**Figure 4:**
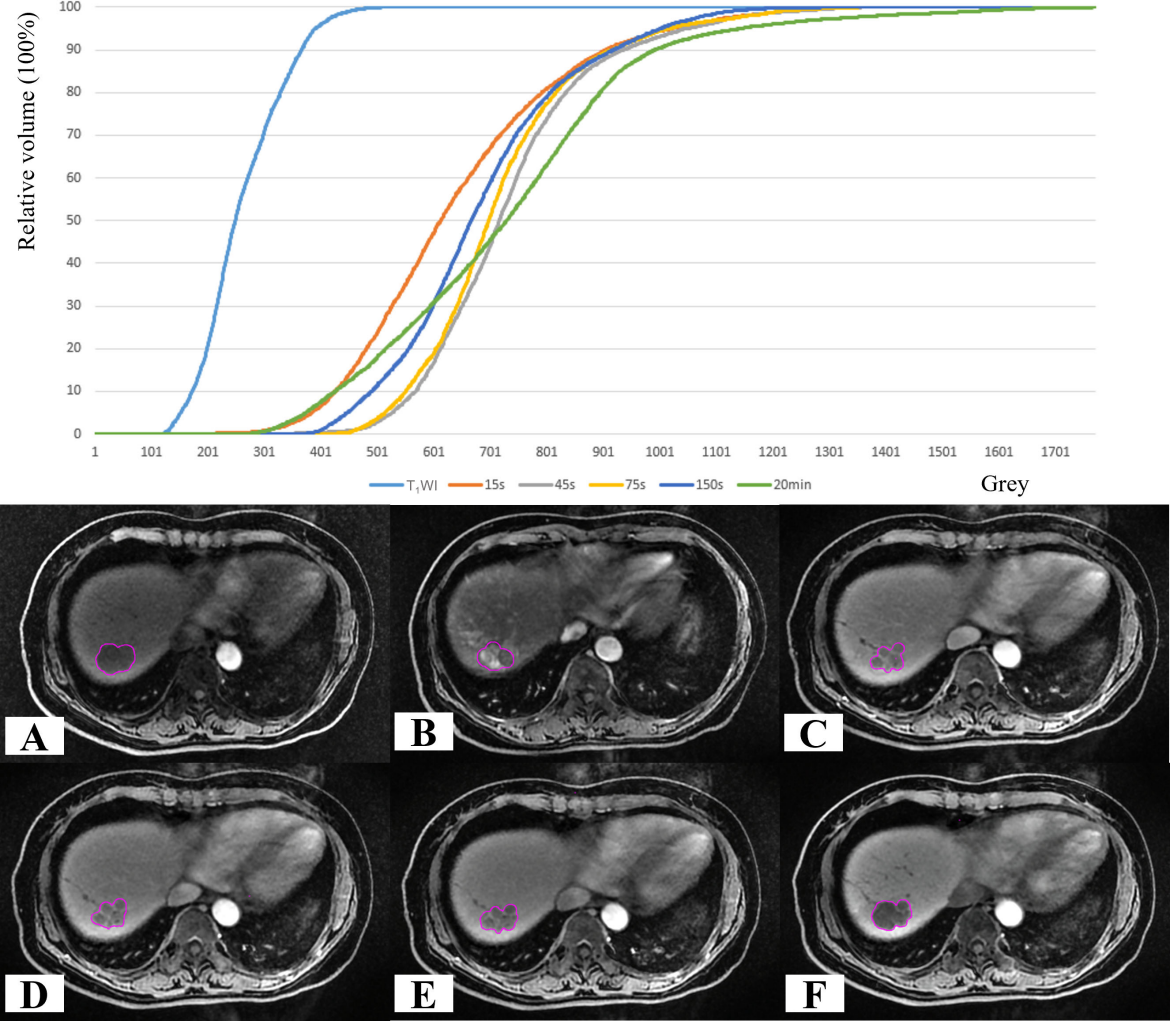
GTV grey histogram of a HCC patient at six phases. **(A)** T_1_WI; **(B–F)** CE-T_1_WI at 15 s, 45 s, 75 s,150 s and 20 min after the intravenous injection of Gd-EOB-DTPA.

Compared with GTV_-20min_, the mean SI of GTV_-T1WI_ and GTV_-15s_~GTV_-150s_ exhibited an increase ranging from -41.19% to 18.09% (**Table 2**). Except for GTV_-150s_, there was a significant difference in the mean SI between GTV_-20min_ and the other GTVs (*p* < 0.05). Furthermore, in pairwise comparisons of the GTV SI, significant differences were observed for all GTVs, except for GTV_-45s_ and GTV_-75s_ (*p* < 0.05) (**Figure 4**).

#### Comparison of mean SI of liver tissue across different phases

The mean SI of the liver tissue exhibited an increasing trend, with the most significant increase observed from CE-T_1_WI_-15s_ to CE-T_1_WI_-45s_. There was no significant change from CE-T_1_WI_-45s_ to CE-T_1_WI_-150s_. However, a slight yet significant increase was observed from CE-T_1_WI_-150s_ to CE-T_1_WI_-20min_ (**Figure 3**). The mean SI of liver tissue reached its maximum at CE-T_1_WI_-20min_, recorded as 651.64 ± 357.75, and its minimum at T_1_WI, with a value of 256.21 ± 88.29 (**Table 2**). Notably, compared with CE-T_1_WI_-20min_, the mean SI of liver tissue decreased by 5.27~55.87% on T_1_WI and CE-T_1_WI_-15s_~CE-T_1_WI_-150s_ (**Table 2**).

**Figure 5:**
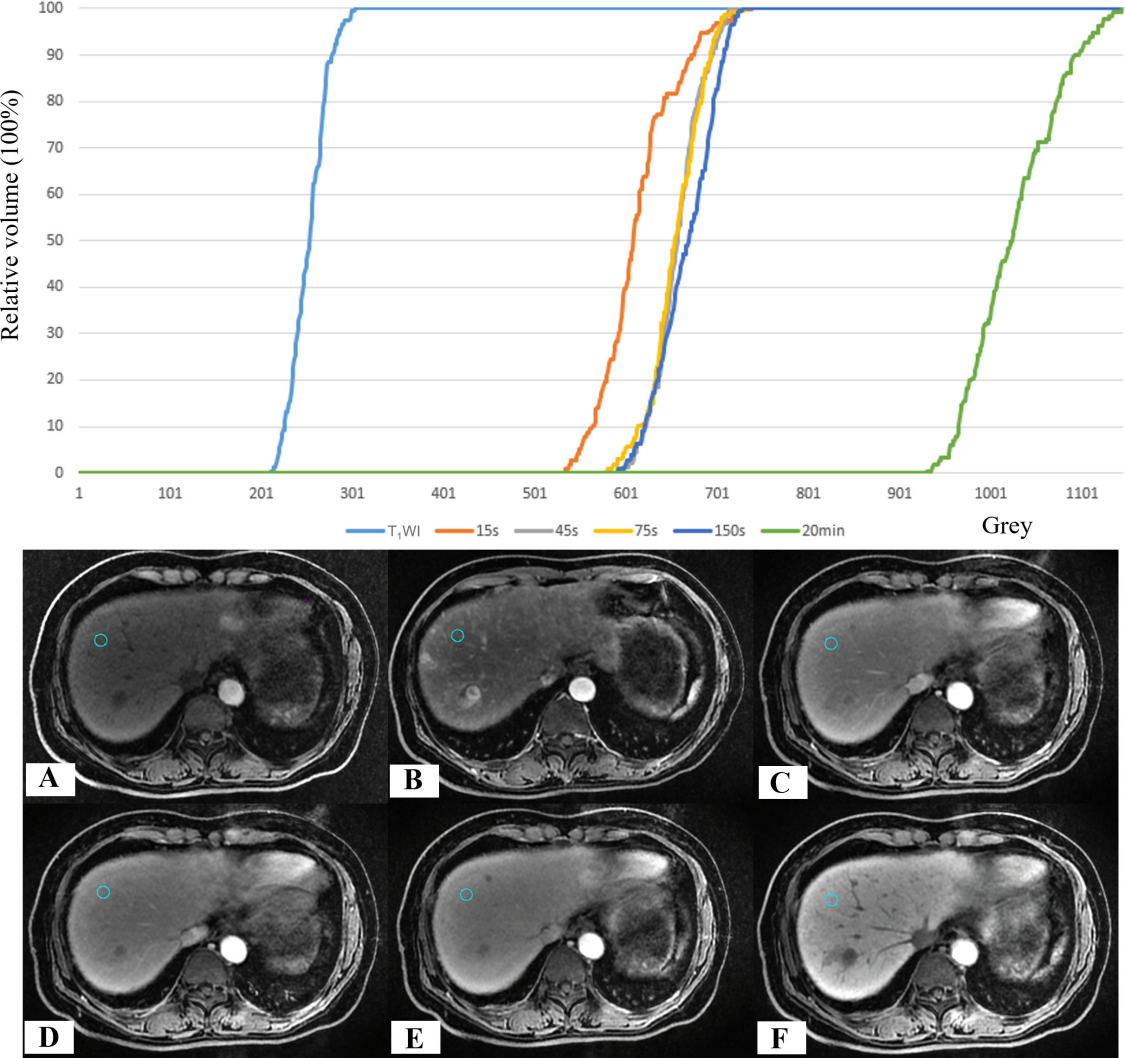
Grey histogram of liver tissue in a HCC patient at six phases. **(A)** T_1_WI; **(B–F)** CE-T_1_WI at 15 s, 45 s, 75 s,150 s and 20 min after the intravenous injection of Gd-EOB-DTPA.

The difference in the mean SI between CE-T_1_WI_-20min_ and the other phases was significant (*p* < 0.05) (**Figure 5**). In the pairwise comparison of the SI of liver tissue across different phases, the mean SI between the other phases showed significant differences (*p* < 0.05), except for CE-T_1_WI_-45s_ and CE-T_1_WI_-75s_; CE-T_1_WI_-45s_ and CE-T_1_WI_-150s_; and CE-T_1_WI_-75s_ and CE-T_1_WI_-150s_.

#### Comparison of SI contrast across different phases

Following the injection of Gd-EOB-DTPA, the SI contrast between HCC and liver tissue increased progressively over time, with the most significant increase observed between CE-T_1_WI_-150s_ and CE-T_1_WI_-20min_ (**Figure 3**). The SI contrast was highest at CE-T_1_WI_-20min_, with a value of 0.29 ± 0.16, whereas it was the lowest at CE-T_1_WI_-15s_, with a value of 0.05 ± 0.19 (**Table 2**). Additionally, significant differences were observed in the SI contrast between CE-T_1_WI_-20min_ and the other phases (*p* < 0.05).

### Comparison of different GTV volumes

**Figure 6:**
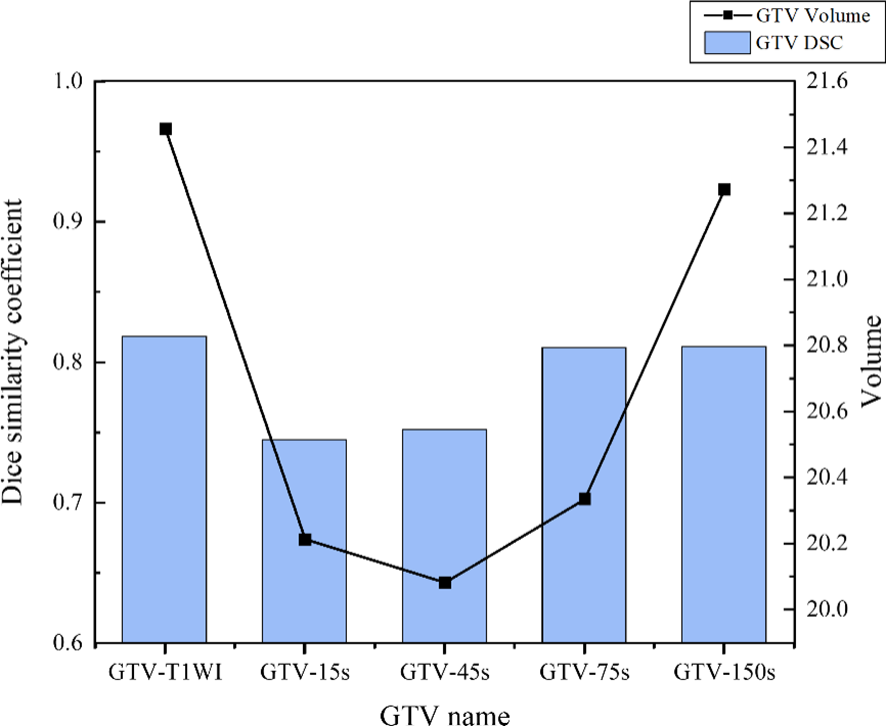
Schematic diagram of different GTVs and DSC changes.

**Figure 7:**
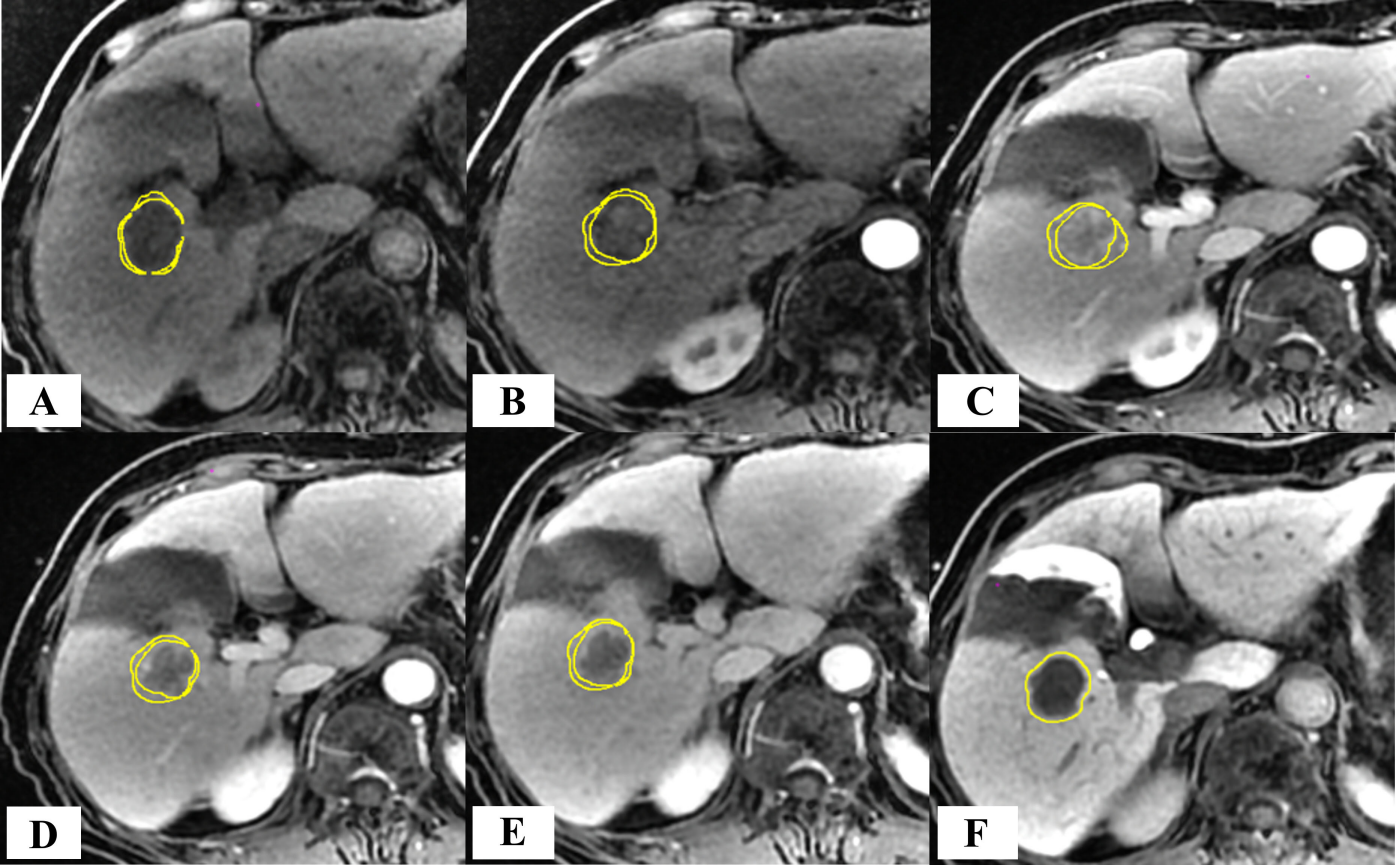
Schematic diagram of the volume difference between GTV across different MR phases in comparison to the GTV_-20min_ of a patient with HCC. **(A)** T_1_WI; **(B–E)** CE-T_1_WI acquired at 15 s, 45 s, 75 s, and 150 s after the intravenous injection of Gd-EOB-DTPA; **(F)** GTV_-20min_ volume diagram.

After injection of Gd-EOB-DTPA, the GTV volumes initially decreased and then gradually increased over time. GTV_-20min_ exhibited the largest volume, with an average of 22.80 ± 18.57 cm^3^, whereas GTV_-45s_ demonstrated the smallest volume, averaging 20.08 ± 18.78 cm^3^ (**Figures 6**, **7**).

Compared with GTV_-20min_, GTV_-T1WI_ exhibited the smallest volume reduction rate, with an average of 6.73 ± 16.25% (*p*>0.05). Conversely, GTV_-45s_ showed the largest volume reduction rate, with an average of 19.35 ± 17.14% (*p* < 0.05) (**Table 3**). Statistical analysis revealed significant volume differences between GTV_-20min_ and GTV_-15s_, GTV_-45s_, and GTV_-75s_ (*p* < 0.05).

**Table 3:** Summary of different GTV volumes, volume differences, and volume reduction rates compared with GTV_-20min_.

GTV name	Volume (cm^3^)	Volume difference (cm^3^)	Volume reduction rate (%)	DSC	*p-*value
**GTV_-T1WI_**	**21.46 ± 18.48**	**1.35 ± 4.91**	**6.73 ± 16.25**	**0.819 ± 0.050**	**0.231**
GTV_-15s_	20.21 ± 19.49	2.59 ± 4.04	17.56 ± 17.63	0.745 ± 0.097	0.005
GTV_-45s_	20.08 ± 18.78	2.72 ± 3.52	19.35 ± 17.14	0.752 ± 0.195	0.003
GTV_-75s_	20.33 ± 18.71	2.47 ± 4.00	15.20 ± 20.30	0.810 ± 0.086	0.022
GTV_-150s_	21.27 ± 19.84	1.53 ± 4.89	12.49 ± 18.33	0.811 ± 0.083	**0.102**
GTV_-20min_	22.80 ± 18.57	\	\	\	\

*p*-values were determined using results from the Wilcoxon test for volume comparison between the other GTV and GTV_-20min_.

### Comparison of the shapes of different GTVs compared with GTV_-20min_

**Figure 8:**
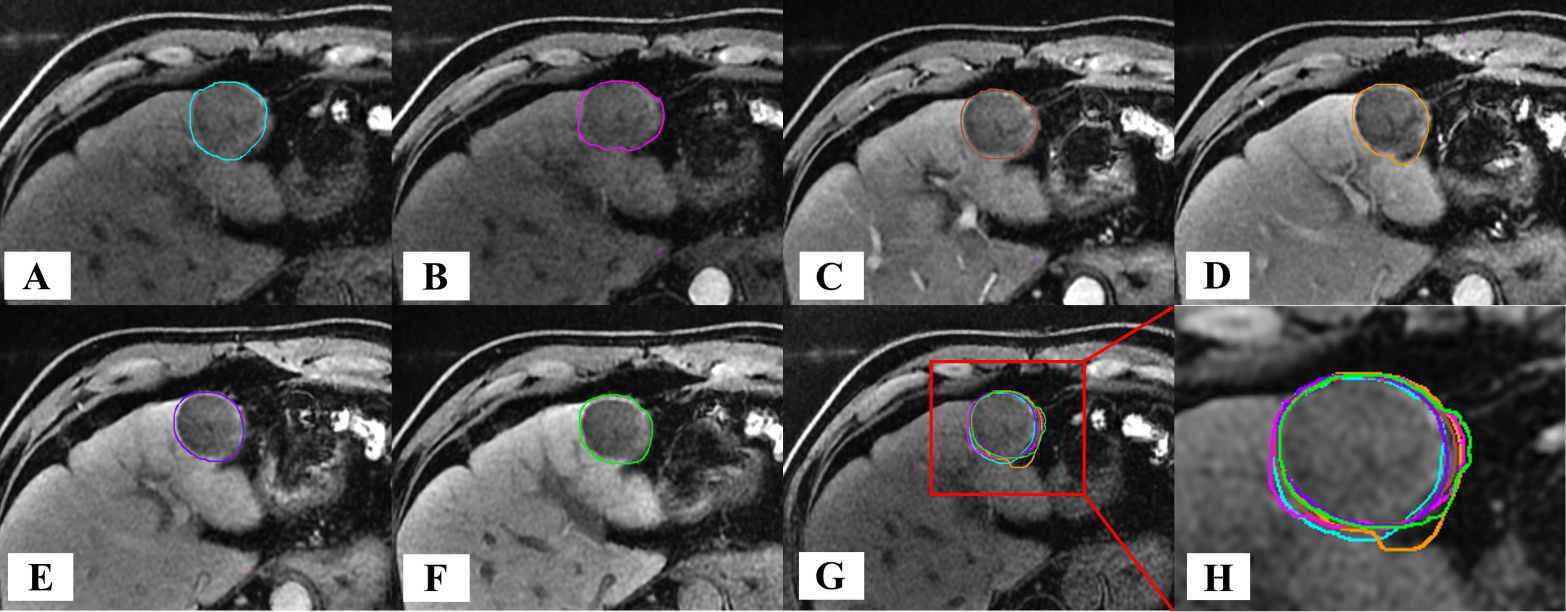
Comparison of GTV shapes across different phases of MR in a patient with HCC. **(A)** GTV shape determination on T_1_WI; **(B–F)** GTV shape determination at 15 s, 45 s, 75 s, 150 s, and 20 min after the intravenous injection of Gd-EOB-DTPA; **(G)** Determination display of six temporal phases on T_1_WI; **(H)** Enlarged display of GTV shape determination.

Compared with GTV_-20min_, the shape and volume change trends of GTVs were generally consistent. The DSC values for GTV_-T1WI_ to GTV_-150s_ were 0.819 ± 0.050, 0.745 ± 0.097, 0.752 ± 0.195, 0.810 ± 0.086, and 0.811 ± 0.083, respectively (**Figures 6**, **8**), with GTV_-T1WI_ exhibiting the largest DSC and GTV_-15s_ showing the smallest DSC.

## Discussion

Accurate determination of the GTV is crucial for improving the efficacy of HCC radiotherapy (3, 8). Therefore, it is pivotal to precisely display and determine the boundary between the tumor and surrounding liver tissue (3). MRI offers superior soft tissue resolution to CT, making it particularly effective for visualizing tumor boundaries (4). Based on previous studies, imaging performed 45s after ECA injection has demonstrated significant advantages in determining tumor volume and shape (9). However, the application of Gd-EOB-DTPA, a hepatobiliary-specific MR contrast agent, in defining the boundaries of HCC GTV has been scarcely investigated. This study aims to evaluate the impact of Gd-EOB-DTPA CE-MRI at different phases on HCC boundary visualization and to provide a theoretical foundation for selecting the optimal phase for GTV determination using Gd-EOB-DTPA CE-MRI.

The difference in blood supply between HCC and liver tissue serves as the foundation for CE-MRI using Gd-EOB-DTPA. The extent and pattern of enhancement observed between lesions and liver tissue following the injection of Gd-EOB-DTPA typically correlates with factors such as perfusion, diffusion level of contrast agent, and expression of the hepatocyte membrane transport proteins OATP1B1 and OATP1B3 (10–13). Notably, Gd-EOB-DTPA exhibits the characteristics of both an ECA and hepatobiliary-specific contrast agent, making it valuable for imaging applications that require visualization of both the vascular and hepatobiliary phases (14). Specifically, the early stages after the injection of Gd-EOB-DTPA exhibit characteristics typical of ECAs. This phase aids in assessing the blood supply to the lesion. Subsequently, during the hepatobiliary phase, the contrast agent demonstrates hepatobiliary-specific characteristics. This phase reflects the presence or absence of liver function and facilitates the visualization of lesions (6).

In this study, the mean SI of both liver tissue and HCC increased most rapidly from 15 s to 45 s after contrast agent injection, which is consistent with the findings by Hamm et al. (15). The injection of an early contrast agent exhibits the characteristics of ECAs by shortening the tissue T_1_ relaxation time (16). At 60 s after injection, hepatocytes begin to absorb Gd-EOB-DTPA, which therefore gradually increases the SI of the liver tissue (6). At 150 s after injection, hepatobiliary-specific contrast agent characteristics start to emerge. As the contrast agent enters the extracellular space from the blood vessels, hepatocytes with normal liver function begin to take up Gd-EOB-DTPA. The SI of the liver tissue, which is influenced by both hepatocytes and the extracellular space contrast agent, increases noticeably (17, 18). Specifically, in HCC, Gd-EOB-DTPA gradually leaves the blood circulation and lacks normal hepatocyte uptake function, leading to a significant decrease in SI (17, 18).

Due to the lower dosage of Gd-EOB-DTPA compared to ECAs, the arterial phase enhancement of HCC was not significant, resulting in a small difference in SI between the tumor and liver tissue, which aligns with the findings of Son et al. (5). As the contrast agent in HCC gradually flows out with the blood, the amount of contrast agent in liver tissue gradually increases. The discrepancy in the SI is heightened between the two tissues, making the boundary between the lesion and liver tissue increasingly distinct. In the present study, the SI contrast was most significantly increased between 150 s and 20 min. Specifically, the SI contrast at 150 s was 37.93% lower than that at 20 min. This was attributed to the contrast agent entering the hepatobiliary phase 20 min after injection, during which the liver tissue re-uptakes Gd-EOB-DTPA (10–12). Consequently, there was a significant increase in SI, compared with the SI at 150 s. However, HCC without hepatocyte function no longer takes up contrast agents, leading to minimal changes in SI (10). Therefore, the contrast between the lesion and surrounding liver tissue was improved, and boundary imaging was clearer at 20 min. This enhancement is beneficial for accurately determining the GTV.

To precisely determine the HCC GTV, it is essential to not only ensure high contrast between the tumor and liver tissue but also to ensure adequate tumor imaging. Due to the dynamic changes in the penetration and outflow of contrast agents, most scholars advocate the use of multi-temporal imaging to fully visualize tumor tissues, consequently enhancing the accuracy of GTV determination (19, 20). However, after understanding the diffusion level of the contrast agent, blood perfusion characteristics, and hepatocyte specificity of Gd-EOB-DTPA, we believe that it is unnecessary to fuse multi-temporal images to determine the GTV based on Gd-EOB-DTPA.

Upon comparing the GTV volumes across different phases, it was observed that the maximum volume was observed on the images acquired 20 min after Gd-EOB-DTPA injection. On the one hand, in HCC, 75% of the blood supply originates from newly generated tumor blood vessels, which possess incomplete and highly permeable basement membranes. Most of the contrast agents have already diffused out of the lesion by 20 min, and the tumor tissue lacking hepatocyte function no longer takes up the contrast agent. On the other hand, the liver tissue has sufficient time to take up the contrast agent and exhibits hepatobiliary phase characteristics. These two mechanisms synergistically resulted in the largest observed GTV.

The volumes of GTV_-15s_ to GTV_-75s_ were 15.20~19.35% smaller than GTV_-20min_, possibly caused by an imbalance in the penetration and outflow of contrast agents in the HCC, leading to insufficient HCC imaging. The lack of a significant difference in volume and shape between GTV_-T1WI_ and GTV_-20min_ may be attributed to the low contrast between the tumor and liver tissue in the absence of the contrast agent. This leads to unclear imaging of the tumor boundary and, consequently, a larger volume. However, due to significant determination errors, its use as the preferred sequence for HCC GTV determination is not recommended.

Based on the CE-T_1_WI images, the longer the time after the Gd-EOB-DTPA injection was completed, the smaller the difference in volume and shape between the GTV and GTV_-20min_. This demonstrates the significance of contrast agent penetration and outflow in HCC imaging. In addition, a small number of HCCs exhibited hypovascularity, with minimal apparent enhancement in the arterial phase. However, determining HCCs based on 20-min CE-T_1_WI images can help decrease the likelihood of missed diagnoses (19, 21).

This study’s innovation lies in utilizing the hepatobiliary-specific contrast agent Gd-EOB-DTPA CE-MRI to determine the HCC GTV. It quantitatively analyzed the differences in HCC GTV determination across different MR phases using Gd-EOB-DTPA, confirming its feasibility in determining HCC GTV for radiotherapy. The primary limitation is the long scanning time required for multiple sequences. To ensure image clarity, high requirements are placed on a patient’s respiratory coordination, resulting in reduced patient tolerance. However, this study excluded images with scanning durations exceeding 20 min. In addition, the small sample size is another limitation of this study. The goal of this study was to verify the feasibility of Gd-EOB-DTPA CE-MRI in determining GTV in radiotherapy. Although the number of subjects was small, the image quality was high. In the future, we will expand the sample size for multi-center promotion.

In summary, when Gd-EOB-DTPA CE-MRI was used to determine the GTV, the CE-T_1_WI sequence with a phase >20 min after injection had apparent advantages in displaying the GTV boundaries and differences in SI. Therefore, it is recommended as the scanning sequence for determining the GTV.

## Data Availability

All data obtained during the current study are available from the corresponding author on reasonable request.

## References

[1] Brown ZJ Tsilimigras DI Ruff SM Mohseni A Kamel IR Cloyd JM . Management of hepatocellular carcinoma: A review. JAMA Surg. (2023) 158:410–20. doi: 10.1001/jamasurg.2022.7989, PMID: 36790767

[2] Jemal A Ward EM Johnson CJ Cronin KA Ma J Ryerson B . Annual report to the nation on the status of cancer, 1975-2014, featuring survival. J Natl Cancer Inst. (2017) 109. doi: 10.1093/jnci/djx030, PMID: 28376154 PMC5409140

[3] Cheung AL Zhang L Liu C Li T Cheung AH Leung C . Evaluation of multisource adaptive MRI fusion for gross tumor volume delineation of hepatocellular carcinoma. Front Oncol. (2022) 12:816678. doi: 10.3389/fonc.2022.816678, PMID: 35280780 PMC8913492

[4] Xiao H Ni R Zhi S Li W Liu C Ren G . A dual-supervised deformation estimation model (DDEM) for constructing ultra-quality 4D-MRI based on a commercial low-quality 4D-MRI for liver cancer radiation therapy. Med Phys. (2022) 49:3159–70. doi: 10.1002/mp.15542, PMID: 35171511 PMC9200368

[5] Son J Hwang SH Park S Han K Chung YE Choi JY . Imaging features of hepatocellular carcinoma: quantitative and qualitative comparison between MRI-enhanced with Gd-EOB-DTPA and Gd-DTPA. Invest Radiol. (2019) 54:494–9. doi: 10.1097/RLI.0000000000000562, PMID: 31094878

[6] Murakami T Sofue K Hori M . Diagnosis of hepatocellular carcinoma using Gd-EOB-DTPA MR imaging. Magn Reson Med Sci. (2022) 21:168–81. doi: 10.2463/mrms.rev.2021-0031, PMID: 34421091 PMC9199982

[7] Chen Y Gong G Wang Y Liu C Su Y Wang L . Comparative evaluation of 4-dimensional computed tomography and 4-dimensional magnetic resonance imaging to delineate the target of primary liver cancer. Technol Cancer Res Treat. (2021) 20:15330338211045499. doi: 10.1177/15330338211045499, PMID: 34617855 PMC8504652

[8] Witt JS Rosenberg SA Bassetti MF . MRI-guided adaptive radiotherapy for liver tumours: visualising the future. Lancet Oncol. (2020) 21:e74–82. doi: 10.1016/S1470-2045(20)30034-6, PMID: 32007208

[9] Meng K Gong G Liu R Du S Wang R Yin Y . Determining the gross tumor volume for hepatocellular carcinoma radiotherapy based on multi-phase contrast-enhanced magnetic resonance imaging. Clin Transl Radiat Oncol. (2025) 50:100877. doi: 10.1016/j.ctro.2024.100877, PMID: 39529652 PMC11550759

[10] Narita M Hatano E Arizono S Miyagawa-Hayashino A Isoda H Kitamura K . Expression of OATP1B3 determines uptake of Gd-EOB-DTPA in hepatocellular carcinoma. J Gastroenterol. (2009) 44:793–8. doi: 10.1007/s00535-009-0056-4, PMID: 19404564

[11] Kitao A Zen Y Matsui O Gabata T Kobayashi S Koda W . Hepatocellular carcinoma: signal intensity at gadoxetic acid-enhanced MR Imaging–correlation with molecular transporters and histopathologic features. Radiology. (2010) 256:817–26. doi: 10.1148/radiol.10092214, PMID: 20663969

[12] Tsuboyama T Onishi H Kim T Akita H Hori M Tatsumi M . Hepatocellular carcinoma: hepatocyte-selective enhancement at gadoxetic acid-enhanced MR imaging–correlation with expression of sinusoidal and canalicular transporters and bile accumulation. Radiology. (2010) 255:824–33. doi: 10.1148/radiol.10091557, PMID: 20501720

[13] Van Rosmalen BV Visentin M Furumaya A Van Delden OM Kazemier G Van Gulik TM . Association between gadoxetic acid-enhanced magnetic resonance imaging, organic anion transporters, and farnesoid X receptor in benign focal liver lesions. Drug Metab Dispos. (2024) 52:118–25. doi: 10.1124/dmd.123.001492, PMID: 38050024

[14] Vogl TJ Kümmel S Hammerstingl R Schellenbeck M Schumacher G Balzer T . Liver tumors: comparison of MR imaging with Gd-EOB-DTPA and Gd-DTPA. Radiology. (1996) 200:59–67. doi: 10.1148/radiology.200.1.8657946, PMID: 8657946

[15] Hamm B Staks T Mühler A Bollow M Taupitz M Frenzel T . Phase I clinical evaluation of Gd-EOB-DTPA as a hepatobiliary MR contrast agent: safety, pharmacokinetics, and MR imaging. Radiology. (1995) 195:785–92. doi: 10.1148/radiology.195.3.7754011, PMID: 7754011

[16] Li J Wang J Lei L Yuan G He S . The diagnostic performance of gadoxetic acid disodium-enhanced magnetic resonance imaging and contrast-enhanced multi-detector computed tomography in detecting hepatocellular carcinoma: a meta-analysis of eight prospective studies. Eur Radiol. (2019) 29:6519–28. doi: 10.1007/s00330-019-06294-6, PMID: 31250172

[17] Hope TA Fowler KJ Sirlin CB Costa EA Yee J Yeh BM . Hepatobiliary agents and their role in LI-RADS. Abdom Imaging. (2015) 40:613–25. doi: 10.1007/s00261-014-0227-5, PMID: 25287679

[18] Yao WW Zhang HWMYP Lee JM Lee RT Wang YL . Comparison of the ability of gadobenate dimeglumine and gadolinium ethoxybenzyl dimeglumine to display the major features for noninvasively diagnosing hepatocellular carcinoma according to the LI-RADS 2018v. Technol Cancer Res Treat. (2024) 23:15330338241260331. doi: 10.1177/15330338241260331, PMID: 38860337 PMC11168249

[19] Hong TS Bosch WR Krishnan S Kim TK Mamon HJ Shyn P . Interobserver variability in target definition for hepatocellular carcinoma with and without portal vein thrombus: radiation therapy oncology group consensus guidelines. Int J Radiat Oncol Biol Phys. (2014) 89:804–13. doi: 10.1016/j.ijrobp.2014.03.041, PMID: 24969794 PMC4285340

[20] Kim YS Kim JW Yoon WS Kang MK Lee IJ Kim TH . Interobserver variability in gross tumor volume delineation for hepatocellular carcinoma: Results of Korean Radiation Oncology Group 1207 study. Strahlenther Onkol. (2016) 192:714–21. doi: 10.1007/s00066-016-1028-2, PMID: 27538775

[21] Golfieri R Renzulli M Lucidi V Corcioni B Trevisani F Bolondi L . Contribution of the hepatobiliary phase of Gd-EOB-DTPA-enhanced MRI to Dynamic MRI in the detection of hypovascular small (≤ 2 cm) HCC in cirrhosis. Eur Radiol. (2011) 21:1233–42. doi: 10.1007/s00330-010-2030-1, PMID: 21293864

